# Understanding Barriers to Colorectal Cancer Screening in Kentucky

**DOI:** 10.5888/pcd12.140586

**Published:** 2015-06-18

**Authors:** Jennifer Redmond Knight, Sarojini Kanotra, Seth Siameh, Jessica Jones, Becki Thompson, Sue Thomas-Cox

**Affiliations:** Author Affiliations: Sarojini Kanotra, Seth Siameh, Becki Thompson, Sue Thomas-Cox, Kentucky Department for Public Health, Frankfort, Kentucky; Jessica Jones, University of Kentucky, Lexington, Kentucky.

## Abstract

**Introduction:**

Colorectal cancer screening rates have increased significantly in Kentucky, from 35% in 1999 to 66% in 2012. A continued improvement in screening requires identification of existing barriers and implementation of interventions to address barriers.

**Methods:**

The state of Kentucky added a question to the 2012 Kentucky Behavioral Risk Factor Surveillance System survey for respondents aged 50 years or older who answered no to ever having been screened for colorectal cancer by colonoscopy or sigmoidoscopy to assess the reasons why respondents had not been screened. Combined responses constituted 4 categories: attitudes and beliefs, health care provider and health care systems barriers, cost, and other. Prevalence estimates for barriers were calculated by using raking weights and were stratified by race/ethnicity, sex, education, income, and health insurance coverage. Logistic regression estimated odds ratios for barriers to screening.

**Results:**

The most common barriers in all areas were related to attitudes and beliefs, followed by health care provider and systems, and cost. Non-Hispanic whites and respondents with more than a high school education were more likely to choose attitudes and beliefs as a barrier than were non-Hispanic blacks and those with less than a high school education. Respondents with low incomes and with no insurance were significantly more likely to select cost as a barrier. No significant associations were observed between demographic variables and the selection of a health care provider and a health care system.

**Conclusion:**

Barriers related to education, race/ethnicity, income, and insurance coverage should be considered when designing interventions. Expansion of Medicaid and implementation of the Affordable Care Act in Kentucky could have an impact on reducing these barriers.

## Introduction

For more than 10 years, local and statewide public health efforts in Kentucky focused on reducing barriers to colorectal cancer screening. Since 2001, the state has seen a 22% decline in both colorectal cancer incidence and mortality (1). According to the Kentucky Behavioral Risk Factor Surveillance System (KyBRFSS), screening rates for colorectal cancer (CRC) using sigmoidoscopy or colonoscopy increased from 34.7% in 1999 to 63.7% in 2008 (2). Screening rates remained static at 63.7% in 2010 (2). Methodology changes in 2011 influenced the KyBRFSS, and in 2012 the screening rate for sigmoidoscopy or colonoscopy was 65.9% (2,3). Although significant progress has been made, to achieve the national and Kentucky CRC screening objective of 80% by 2018, it is necessary to understand and address the greatest barriers to screening (4,5).

### Disparities in colorectal cancer screening

Despite having effective CRC screening methods available, some subpopulations have not received any type of screening. One study examining Behavioral Risk Factor Surveillance System (BRFSS) 2010 data found that the respondents most likely never to have never been screened are younger (50–59 y), male, and non-Hispanic Asian/Native Hawaiian/Pacific Islander (6). The study showed that screening rates increased with education, and those with lower educational attainment were screened less often for CRC than those with a higher educational attainment (6). A 2012 follow-up study examined the same CRC screening questions from the BRFSS and found that the percentage of blacks and whites that had been screened for CRC were almost equal and were higher than CRC screening rates for other races/ethnicities (7). Those who had never been screened were most often people aged 50 to 64, men, Hispanics, American Indians/Alaska Natives, and people living outside of urban areas (7). This study also showed that the lower the education and income level, the lower the CRC screening rates (7). Additional disparities among those who had never been screened were having no health insurance (55.0%) and no regular health care provider (61.0%) (7).

Kentucky’s screening disparities were similar to those of the United States overall. The most notable Kentucky-specific disparity in CRC screening was related to educational status, and the size of the disparity has increased since 1999 (2,8,9). People with less than a high school education have had consistently lower sigmoidoscopy and colonoscopy screening rates compared with college graduates (8,9). In 1999, people with less than a high school education had a 33.4% CRC screening rate compared with 36.3% of college graduates (9). In 2012, people with less than a high school education had a 55.2% CRC screening rate compared with a 73.5% rate for college graduates (9).

### Barriers and facilitators to colorectal cancer screening

Barriers to CRC screening are complex, intertwined, and related to knowledge, motivation, and ability (10). Fear of the procedure and bowel preparation are common barriers to CRC screening (10,11). Among racially diverse populations with less than a high school education, low income, no health insurance, and no regular health care provider, other barriers to CRC screening are fatalism, religious beliefs, lack of self-worth, sexually related concerns, history of sexual abuse, past negative experiences with screening, and suspicion that a physician may be motivated to recommend the procedure for financial gain (10). Knowledge, perceptions, and beliefs about CRC screening and an individual’s cultural, social, and physical environments influence the decision to undergo preventive screening (12,13). Physicians report that they perceived barriers to CRC screening to be patients' failure to follow through with recommended screening, cost of screening procedures, and lack of insurance coverage (14). Even without individual-level barriers related to knowledge, perceptions, and beliefs or with a physician’s recommendation, people may be unable to obtain recommended screenings because of structural barriers or inadequate resources (10). According to the KyBRFSS, in 2008 in Kentucky, the greatest barriers to CRC screening with sigmoidoscopy or colonoscopy were beliefs that screening was not needed, the person had no symptoms, there was no family history of cancer (27%), and screening was not recommended by the person’s doctor (27%) (15).

Knowing one’s risk profile based on family history and susceptibility for disease facilitates health behavior (13,16). However, multiple studies demonstrate that CRC screening increases when a physician or other clinician recommends the test to their patients, and one study attributes 20% of the effect size to patient–provider communication (10,16–19).

Although progress has been made in the past decade to increase CRC screening rates in Kentucky, certain population groups have not benefitted from early detection. To increase CRC screening in Kentucky among disparate populations, specifically those with low educational attainment, it is necessary to understand barriers to screening. This study aimed to gather population-level data on the individual-, social-, environmental-, and systems-level barriers specific to Kentuckians that prevent them from obtaining CRC screening. Our objective was to provide information for state, regional, and local-level partners throughout Kentucky and in states with similar populations that can be used to develop and implement effective strategies to reduce these barriers and increase CRC screening rates.

## Methods

The KyBRFSS is a statewide telephone health survey jointly sponsored by the Centers for Disease Control and Prevention (CDC) and the Kentucky Department for Public Health (KDPH), conducted annually since 1985. KyBRFSS data contribute to CDC’s Behavioral Risk Factor Surveillance System (BRFSS), and similar surveys are conducted in every state, the District of Columbia, and in several US territories. Randomly selected, noninstitutionalized adults who live in a household with a telephone are candidates for the survey. Participation in the survey is strictly voluntary. Personal identifying information, such as a person’s name or address, is not collected.

**Figure 1:**
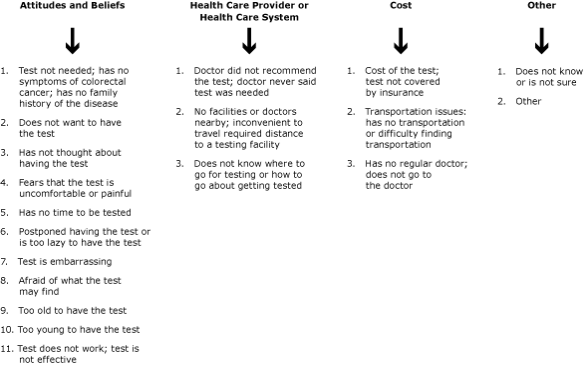
Nineteen reasons cited by respondents aged 50 years or older who answered no to question assessing barriers to colorectal cancer screening: “Have you ever been screened for colorectal cancer by sigmoidoscopy or colonoscopy,” in the 2012 Kentucky Behavioral Risk Factor Surveillance System Survey.

**Figure 2:**
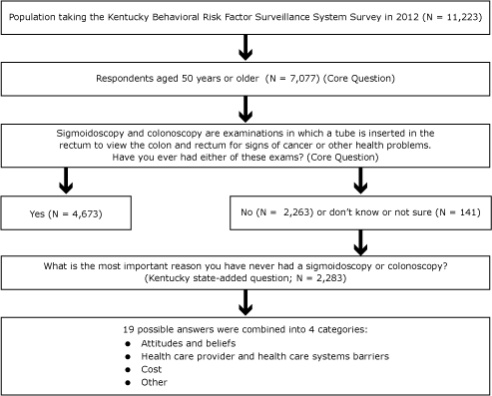
Paradigm used to identify Kentuckians aged 50 years or older never screened for colorectal cancer via colonoscopy or sigmoidoscopy, from survey question about barriers to colorectal cancer screening in 2012 Kentucky Behavioral Risk Factor Surveillance System.

A Kentucky-added CRC screening question related to barriers was added to the 2008 and 2012 KyBRFSS for respondents aged 50 years or older who answered no to ever having been screened by sigmoidoscopy or colonoscopy (n = 2,263). In 2012, 19 potential responses to this added question were grouped into 4 categories of barriers: 1) attitudes and beliefs, 2) health care provider and health care systems barriers, 3) cost, and 4) other (Figure 1). Figure 2 describes the process and number of respondents for this 2012 question. In addition to the 2,263 who responded no, 141 responded “don’t know/not sure.” Twenty of the 141 “don’t know/not sure” respondents participated in the question about barriers. The total respondents for the barrier question was 2,283 (2,263 + 20).

In 2011, the BRFSS, in collaboration with CDC and the Commonwealth of Kentucky, instituted a change in BRFSS methods (3). Changes in data collection were based on an ever-growing population of cellular phone users, many of whom do not use landline telephones and the introduction of “raking,” (iterative proportional fitting), a new weighting procedure (20). Raking allows for the inclusion of additional demographic variables in the weighting process, thereby ensuring prevalence estimates that are more representative of the population from which the data are gathered (3,20). Because of these significant changes, direct comparisons of 2008 data with 2012 data are not possible.

For 2012 data, calculated prevalence estimates for barriers used raking weights were stratified by the following parameters: race/ethnicity, sex, education, income, and health insurance coverage. Logistic regression was used to estimate odds ratios (ORs) for barriers to screening. All statistical analyses were conducted with SAS version 9.3 (SAS Institute Inc).

## Results

The greatest disparities for never having a sigmoidoscopy or colonoscopy were educational status (those with less than a high school education), race/ethnicity (non-Hispanic black), income (<$25,000/y), sex (male), health insurance status (not having any) (Table 1). Barriers to screening varied significantly by demographic characteristics. The most common barriers in all areas were related to attitudes and beliefs (62.4%; 95% confidence interval [CI], 59.2%–65.5%) followed by health care provider and health care systems (15.9%; 95% CI, 13.5–18.2) and cost (11.7%; 95% CI, 9.5%–13.9%). (Table 2).

**Table 1:** Screening for Colorectal Cancer Via Sigmoidoscopy or Colonoscopy, Respondents (N = 2,283) to Kentucky Behavioral Risk Factor Surveillance System Survey, 2012

Characteristic	Yes	No
	% (95% Confidence Interval)	
**All adults aged ≥50y**	65.9 (64.2–67.6)	34.1 (32.4–35.8)
**Sex**		
Male	63.3 (60.4–66.2)	36.7 (33.8–39.6)
Female	68.2 (66.1–70.2)	31.8 (29.8–33.9)
**Race/ethnicity**		
Non-Hispanic white	66.4 (64.6–68.2)	33.6 (31.8–35.4)
Non-Hispanic black	63.4 (55.2–71.6)	36.6 (28.4–44.8)
**Education**		
<High school	55.2 (50.4–60.0)	44.8 (40.0–49.6)
≥High school	68.8 (67.0–70.6)	31.2 (29.4–33.0)
**Income, $**		
≤24,999	58.6 (55.4–61.8)	41.4 (38.2–44.6)
25,000–49,999	69.6 (66.1–73.0)	30.4 (27.0–33.9)
≥50,000	70.1 (67.0–73.2)	29.9 (26.8–33.0)
**Health insurance**		
Yes	69.5 (67.8–71.2)	30.5 (28.8–32.2)
No	30.0 (24.2–35.8)	70.0 (64.2–75.8)

**Table 2:** Barriers to Colorectal Cancer Screening, by Demographic Characteristic, Respondents (N = 2,283) to Kentucky Behavioral Risk Factor Surveillance System Survey, 2012

Characteristic	Attitudes and Beliefs	Health Care Provider and Health Care Systems Barriers	Cost	Other Barriers
	% (95% Confidence Interval)			
**All adults aged ≥ 50 y**	62.4 (59.2–65.5)	15.9 (13.5–18.2)	11.7 (9.5–13.9)	10.0 (8.1–12.0)
**Sex**				
Male	60.7 (55.6–65.7)	15.6 (11.8–19.3)	13.8 (9.8–17.8)	10.0 (6.9–13.0)
Female	64.0 (60.3–67.7)	16.2 (13.4–19.0)	9.7 (7.6–11.7)	10.1 (7.6–12.7)
**Race/ethnicity**				
Non-Hispanic white	64.5 (61.3–67.7)	14.9 (12.7–17.1)	10.9 (8.6–13.2)	9.6 (7.6–11.6)
Non-Hispanic black	44.3 (31.1–57.5)	15.9 (6.0–25.7)	21.2 (8.2–34.1)	18.6 (6.3–31.0)
**Education**				
<High school	53.4 (46.1–60.8)	16.5 (10.8–22.1)	15.4 (9.5–21.2)	14.7 (9.6–19.9)
≥High school	65.9 (62.6–69.1)	15.4 (13.1–17.8)	10.4 (8.2–12.6)	8.3 (6.4–10.2)
**Income, $**				
≤24,999	55.9 (50.8–60.9)	16.7 (13.0–20.5)	16.9 (13.1–20.6)	10.5 (7.4–13.7)
25,000–49,999	62.0 (55.0–68.9)	17.6 (12.9–22.2)	14.5 (8.1–20.9)	5.9 (2.9–9.0)
≥50,000	69.5 (63.7–75.4)	15.5 (10.3–20.6)	4.7 (2.0–7.5)	10.3 (6.7–13.9)
**Health insurance**				
Yes	66.8 (63.5–70.1)	16.6 (14.0–19.3)	5.0 (3.6–6.5)	11.5 (9.2–13.9)
No	43.0 (34.9–51.1)	12.5 (7.7–17.3)	40.8 (32.5–49.1)	3.7 (1.3–6.0)

Respondents with more than a high school education were more likely to identify attitude and beliefs as a barrier than those with less than a high school education. Non-Hispanic black respondents were 44% less likely to choose attitudes and beliefs as a barrier than white respondents. Respondents with low income were also significantly less likely to select attitude and beliefs than those with high income (Table 3). Associations between the selection of health care provider and health care system and the demographic variables (race/ethnicity, sex, education, income, or health insurance coverage) were not significant (Table 3). Only 5% of respondents with health insurance stated that cost was a barrier versus 41% of those with no health insurance (Table 2). Respondents with low income were significantly more likely to select cost as a barrier than respondents with an annual income of $50,000 or more (Table 3).

**Table 3:** Demographic Variables Associated With Barriers to Colorectal Cancer Screening, Respondents (N = 2,283) to Kentucky Behavioral Risk Factor Surveillance System Survey, 2012

Variable	Attitudes and Beliefs	Health Care Providers and Systems	Cost	Other Barriers
Odds Ratio (95% Confidence Interval) [*P* Value]				
**Sex**				
Male	1 [Reference]			
Female	1.15 (0.89–1.50) [.29]	1.05 (0.74–1.48) [.80]	0.67 (0.45–1.01) [.05]	1.02 (0.66–1.57) [.93]
**Race/ethnicity**				
Non-Hispanic white	1 [Reference]			
Non-Hispanic black	0.44 (0.25–0.76) [.003]	1.08 (0.51–2.30) [.85]	2.19 (0.98–4.91) [.06]	2.19 (0.98–4.91) [.08]
**Education**				
<High School	1 [Reference]			
≥High School	1.68 (1.21–2.33) [.002]	0.93 (0.59–1.45) [.74]	0.64 (0.39–1.06) [.08]	0.52 (0.33–0.84) [.008]
**Income, $**				
≤24,999	0.56 (0.39–0.79) [<.001]	1.10 (0.68–1.76) [.70]	4.07 (2.09–7.92) [<.001]	1.03 (0.62–1.71) [.91]
25,000–49,999	0.72 (0.48–1.07) [.10]	1.16 (0.70–1.93) [.55]	3.41 (1.54–7.57 [.003]	0.55 (0.28–1.07) [.08]
≥50,000	1 [Reference]			
**Health insurance**				
Yes	1 [Reference]			
No	0.38 (0.26–0.54) [<.001]	0.72 (0.45–1.15) [.17]	13.0 (8.25–20.48) [<.001]	0.29 (0.15–0.59) [<.001]

## Discussion

Barriers to screening vary significantly on the basis of educational status, race/ethnicity, income, and insurance status and, if addressed, could increase screening. Barriers related to attitude and beliefs were more prevalent among white adults, adults with more than a high school education, and those with annual incomes of $50,000 or more. Cost barriers were more prevalent among black adults, adults with lower education, and those with lower levels of income. To increase CRC screening, interventions should focus on removing the most common barriers for each population group discussed. Regardless of the type of barrier, the most important consideration is that the barriers are removed. Once the specific barriers to screening for each population are removed, screening rates should become the same for all populations. This should then decrease disparities in CRC screening.

This study had limitations. The 2012 KyBRFSS did not gather information about barriers to blood-stool testing (fecal occult blood test [FOBT] and fecal immunochemical test [FIT]), which is known to be effective (21). Comparing barriers among the types of CRC screening would have strengthened the study. It is also unknown whether cognitive testing was performed for the added Kentucky question to ensure survey participants’ comprehension. This question was originally adapted from a state-added question used in New Mexico and Utah (22).

Addressing the complex barriers that prevent people from obtaining CRC screening requires an ecological approach with interventions targeted at individual, interpersonal, relational, institutional, systems, social, and policy levels (13,14). A previous study of CRC screening was conducted in New Mexico by the Clinical Prevention Initiative, a statewide partnership of health care organizations supported by CDC and the New Mexico Department of Health. The study used the BRFSS to survey physicians and a general population to analyze barriers to CRC screening (22). Although this study focused on different screening methods, for example, FOBT or lower endoscopy, the New Mexico results from its state-added 2004 BRFSS barriers question were similar to Kentucky’s responses in 2008 but not in 2012 (22). The most frequently cited reason for never having obtained a CRC screening was lack of physician recommendation, followed by lack of symptoms (22). Lack of physician recommendation and not knowing CRC screening was necessary were also top barriers noted in another study of patient-reported barriers to CRC screening (23).

Although some similarities appear across studies, barriers in this study are not homogenous across demographic groups or even types of CRC screening tests (23). Interventions to increase CRC screening should involve collaboration among relevant organizations to address multiple barriers.

Many educational efforts in Kentucky have focused on addressing barriers related to attitudes and beliefs. Our study results indicate that this approach may reach only the white population with annual incomes above $25,000 and a high school education or more. To address screening among blacks, people with less than a high school education, and people with income below $25,000 a year, cost barriers must also be addressed.

This study’s findings support the need to address cost as a barrier to CRC screening, which is also related to insurance coverage. Among the white, educated (more than a high school education) population, 85.2% were insured. White respondents with less than a high school education had a 72.0% insured rate. Black, educated respondents had a 69.3% insured rate whereas black respondents with less than a high school education had a 74.8% insured rate. Most of the less educated (less than a high school education) white respondents (46.2%) lived in rural Kentucky, and most of the more highly educated white respondents (54.1%) lived in urban Kentucky. For blacks, both the more highly educated and less educated respondents lived primarily in urban Kentucky (74%). Geographic classifications were based on urban–rural continuum codes provided by the US Department of Agriculture (24). With implementation of the Affordable Care Act (ACA) and the enrollment success that the expansion of Medicaid and Kentucky’s state-based exchange, known as kynect, has demonstrated since its implementation, fewer Kentuckians will remain uninsured; these circumstances should mitigate some cost and health insurance barriers (25). One criterion for health plans that fall within ACA guidelines is that they cover preventive services, including colorectal cancer screening, with no copay or deductible. In the past, as a result of loopholes in insurance coverage, patients in Kentucky had out-of-pocket costs when they had a colonoscopy that began as screening and resulted in polyp removal or they had a positive FIT or FOBT that required a colonoscopy. In March 2015, the Kentucky General Assembly passed legislation that was signed by the Governor that requires that all screening colonoscopies be covered without a co-pay or deductible for these previous loopholes in screening coverage (26). Health benefit plans that are renewed on or after January 1, 2016, will no longer be able to impose a deductible or co-pay for patients who have a screening colonoscopy that results in polyp removal or a positive FIT or FOBT that requires a follow-up colonoscopy (26).

Programs need to consider the target population and the most common barriers when developing and tailoring interventions to increase CRC screening. Interventions may be more effective when combining approaches to address multiple barriers, such as attitudes and beliefs, health care provider recommendations *and* access to screening, cost, *and* insurance coverage. By working together to overcome these barriers through multiple interventions and partnerships, Kentucky can continue to make progress toward increase CRC screening.

The Kentucky Cancer Consortium (KCC) will continue to monitor the BRFSS to assess screening rates as efforts are focused on reducing barriers to CRC screening. Current efforts among the KCC member organizations include working with the American Cancer Society to determine how to incorporate new, market-tested messages that resonate with the insured and unwilling populations and working with the American Cancer Society, the Colon Cancer Prevention Project, the KDPH, the Kentucky Cancer Program (KCP), and the University of Kentucky Regional Extension Centers to work with federally qualified community health centers to reduce barriers and increase screening, particularly among blacks, urban residents, and rural whites, especially whites in Appalachian Kentucky. Kentucky is fortunate to have the KCP, a community-based comprehensive cancer program. Through KCP, district cancer councils are bringing together community organizations and partners to better understand what is needed to address barriers in local and regional areas and will implement local and regional approaches to increasing CRC screening, particularly among those with lower education levels. In addition, Kentucky has joined the national *80% by 2018* campaign and plans to use national momentum to reach Kentuckians who may have insurance and have not yet been screened.
